# Sources and Fates of Carbamyl Phosphate: A Labile Energy-Rich Molecule with Multiple Facets

**DOI:** 10.3390/biology7020034

**Published:** 2018-06-12

**Authors:** Dashuang Shi, Ljubica Caldovic, Mendel Tuchman

**Affiliations:** 1 Center for Genetic Medicine Research, Children’s National Medical Center, Washington, DC 20010, USA; LCaldovic@childrensnational.org (L.C.); Mtuchman@cnmc.org (M.T.); 2 Department of Genomics and Precision Medicine, The George Washington University, Washington, DC 20010, USA

**Keywords:** carbamyl phosphate, urea cycle, arginine biosynthesis, pyrimidine biosynthesis, transcarbamylase, carbamate kinase

## Abstract

Carbamyl phosphate (CP) is well-known as an essential intermediate of pyrimidine and arginine/urea biosynthesis. Chemically, CP can be easily synthesized from dihydrogen phosphate and cyanate. Enzymatically, CP can be synthesized using three different classes of enzymes: (1) ATP-grasp fold protein based carbamyl phosphate synthetase (CPS); (2) Amino-acid kinase fold carbamate kinase (CK)-like CPS (anabolic CK or aCK); and (3) Catabolic transcarbamylase. The first class of CPS can be further divided into three different types of CPS as CPS I, CPS II, and CPS III depending on the usage of ammonium or glutamine as its nitrogen source, and whether *N*-acetyl-glutamate is its essential co-factor. CP can donate its carbamyl group to the amino nitrogen of many important molecules including the most well-known ornithine and aspartate in the arginine/urea and pyrimidine biosynthetic pathways. CP can also donate its carbamyl group to the hydroxyl oxygen of a variety of molecules, particularly in many antibiotic biosynthetic pathways. Transfer of the carbamyl group to the nitrogen group is catalyzed by the anabolic transcarbamylase using a direct attack mechanism, while transfer of the carbamyl group to the oxygen group is catalyzed by a different class of enzymes, CmcH/NodU CTase, using a different mechanism involving a three-step reaction, decomposition of CP to carbamate and phosphate, transfer of the carbamyl group from carbamate to ATP to form carbamyladenylate and pyrophosphate, and transfer of the carbamyl group from carbamyladenylate to the oxygen group of the substrate. CP is also involved in transferring its phosphate group to ADP to generate ATP in the fermentation of many microorganisms. The reaction is catalyzed by carbamate kinase, which may be termed as catabolic CK (cCK) in order to distinguish it from CP generating CK. CP is a thermally labile molecule, easily decomposed into phosphate and cyanate, or phosphate and carbamate depending on the pH of the solution, or the presence of enzyme. Biological systems have developed several mechanisms including channeling between enzymes, increased affinity of CP to enzymes, and keeping CP in a specific conformation to protect CP from decomposition. CP is highly important for our health as both a lack of, or decreased, CP production and CP accumulation results in many disease conditions.

## 1. Introduction

Carbamyl phosphate (CP), discovered and synthesized by Jones and Lipmann in 1955 [1], is an interesting compound combining ammonia, carbonate, and phosphate in a single molecule. It is believed that CP may have originated in the prebiotic world providing a source of a carbamyl group for the biosynthesis of many important organic molecules such as pyrimidine and arginine, and the source of a phosphate group to ADP to make ATP, that are crucial for all forms of life [2,3]. It was demonstrated that the synthetic CP is active in donating the carbamyl group to ornithine to form citrulline with enzymes prepared from bacteria and liver. It was also found that the synthetic CP could be the carbamyl donor for aspartate. Further studies confirmed that the synthetic CP is identical to the intermediate formed in the mammalian-citrulline-synthesizing system [4].

**Figure 1:**
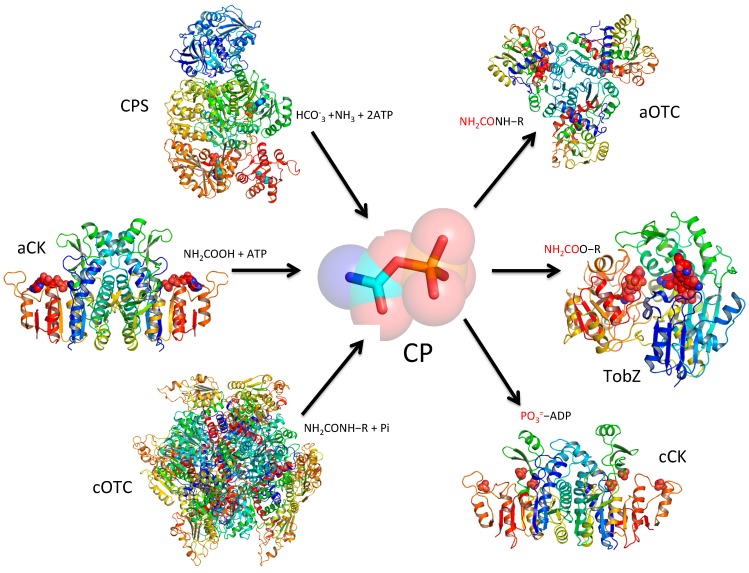
Sources and fates of carbamyl phosphate. Enzymatically, carbamyl phosphate (CP) can be synthesized using three different classes of enzymes: (1) ATP-grasp fold protein based carbamyl phosphate synthetase (CPS) from bicarbonate, ammonia, and two ATP; (2) Amino-acid kinase fold carbamate kinase (CK)-like CPS (anabolic CK or aCK) from carbamate and ATP; and (3) Catabolic transcarbamylase (cOTC) from phosphorolysis of ureido-containing compounds such as citrulline, agmatine, and allantoate. CP can donate a carbamyl group to an amino group using anabolic transcarbamylases, a hydroxyl group using an *O*-transcarbamylase, and even a sulfur group using HypF (the enzyme is not drawn in this figure for clarity). CP can also donate a phosphate group to ADP generating ATP using catabolic carbamate kinase (cCK). The represented enzymes are drawn as cartoon diagrams with the coordinates from Protein Data Bank (CPS, PDB ID: 5dou; aCK, PDB ID: 1e19; cOTC, PDB ID: 1DXH; aOTC, PDB ID 1e9y; TobZ, 3vet; cCK, PDB ID: 2we4).

Enzymatically, three very different classes of enzymes produce CP (Figure 1). The first one, which is broadly distributed from microorganisms to humans, uses a basic ATP-grasp fold enzyme [5] to catalyze three-step formation of CP [6,7]. This process irreversibly consumes two moles of ATP in order to make one mole of CP. Ammonia, required for reaction, could be supplied either externally or internally by the glutaminase domain; it can be either fused to the ATP-grasp fold enzyme or associated with it as a subunit of a heterodimer. The second one, claimed to exist only in microorganisms such as the hyperthermophilic archaea *Pyrococcus abyssi* and *Pyrococcus furiosus* [8,9,10,11,12], employs an amino-acid kinase fold carbamate kinase-like protein to catalyze the reversible reaction of carbamate with ATP to form CP and ADP. The third one, which also exists only in microorganisms such as *Streptococcus mutants*, *Enterococcus faecalis*, lactobacilli, *Giardia intestinalis*, and *Trichomonas vaginalis*, uses catabolic transcarbamylases to generate CP. Since this reaction is thermodynamically unfavorable, the CP generating reaction can only proceed by coupling with carbamate kinase to generate ATP in vivo [13,14].

CP as one of eight key small molecules to power cell metabolism [15] provides the carbamyl group for many biosynthetic pathways. The most well-known pathways are the urea/arginine pathway that produces urea and arginine and the pyrimidine pathway that provides essential nucleotides for DNA synthesis [1,4,16]. However, many antibiotic synthesis pathways also involve the carbamyl group transfer from CP to either amino groups or a hydroxyl group [17,18,19,20,21,22,23]. Furthermore, CP even participates in the [NiFe] hydrogenase maturation by providing the CN group to the [NiFe] cluster. In this process, the hydrogenase pleiotropically acting protein A (HypA) catalyzes a carbamyl transfer reaction from carbamate, which is generated from CP, to the C-terminal cysteine residue of HypE via a carbamyladenylate intermediate [24]. HypE catalyzes an ATP-dependent dehydration to produce a thiocynate group, whose cyano group then eventually becomes the ligand of [NiFe](CN)_2_CO’s active site center for [NiFe] hydrogenase.

This review article focuses on how CP is generated biochemically and enzymatically and how CP is involved in the biosynthesis of many important biological molecules including arginine, pyrimidine, and ATP. CP was last reviewed by Jones in 1963 and many new insights have been obtained about its generation and usage since then. It is an opportune time to summarize many facets of this important small molecule, which is a well-known essential intermediate of pyrimidine and arginine/urea biosynthesis and is highly correlated with human health.

## 2. Production of CP Chemically

Chemically, CP is easily produced by mixing dihydrogen phosphate with cyanate in a solution [1]. In brief, equal moles of dihydrogen phosphate and cyanate are mixed and warmed to 30° for 30 min. Then, the solution is cooled on ice, to which an ice-cold solution of the mixture of lithium hydroxide and perchloric acid is added to adjust to a final pH of 8.3. The precipitate, which consists of potassium perchlorate and lithium phosphate, is removed by filtration. About 90% to 95% pure dilithium CP can be obtained by adding ethanol slowly to the filtrate.

## 3. Production of CP in Biological Systems

### 3.1. Production of CP Using CPS

In biological systems, several enzymes are able to catalyze the formation of CP. Carbamyl phosphate synthetase (CPS) is the most common one; it occurs in nearly all organisms and is a multi-domain protein with about 1500 amino acid residues comprising one or two or more polypeptide chains. Three different types of CPS termed CPS I, CPS II, and CPS III are recognized depending on the nitrogen sources and allosteric activator [25]. CPS I uses only ammonia as the nitrogen-donating substrate and requires *N*-acetyl-glutamate (NAG) as its essential allosteric activator. This enzyme is located in mitochondria and functions as the first step to remove ammonia in the liver via the urea cycle and to make arginine by the combined actions of intestine, which contains CPS1, and the kidney, that makes arginine from citrulline [26,27]. CPS II uses glutamine as its nitrogen-donating substrate and does not require NAG for activity. In humans, CPS II is located in the cytosol as a pyrimidine biosynthesis enzyme and forms a multifunctional complex (termed CAD) with the next two enzymes of the pyrimidine biosynthetic pathway, aspartate transcarbamylase (ATCase) and dihydroorotase (DHO). CPS II is also present in fungi and bacteria. Fungi have two different forms of CPS II, one is pyrimidine-specific, and one is arginine-specific, but bacteria usually only have one CPS II for both pathways. The fungal pyrimidine-specific CPS II is fused to ATCase and active or inactive DHO similar to human multifunctional complex CAD. The fungal arginine-specific and bacterial CPS II is either a heterodimer consisting of glutaminase and synthase subunits, or a single peptide with both subunits fused. CPS III, which is found in invertebrates and fish, is composed of one single peptide and uses glutamine as a nitrogen-donating source and requires NAG for optimal activity [25], suggesting that CPS III is an evolutionary intermediate in the transition from glutamine-dependent CPS II to ammonia-dependent CPS I.

**Figure 2:**
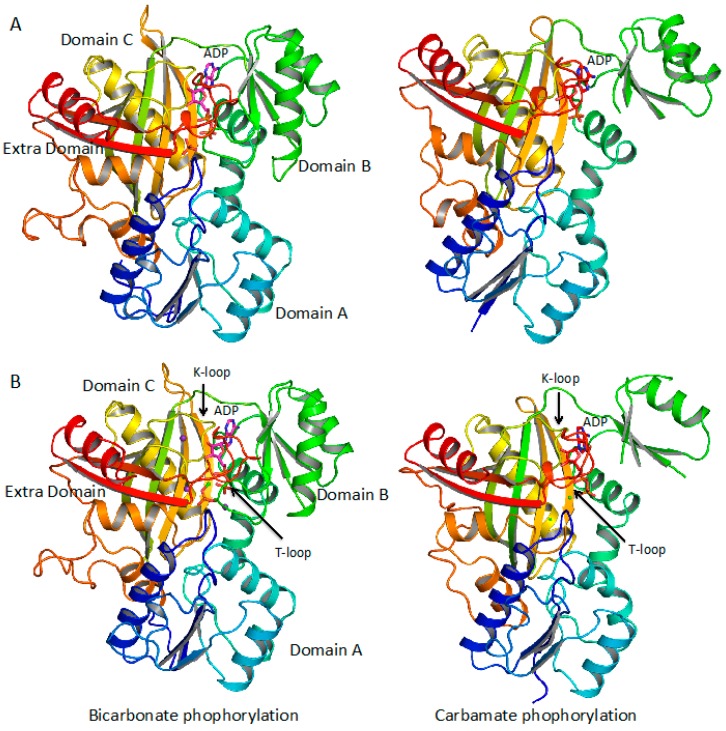
Ribbon diagram of the structure of A1 (bicarbonate phosphorylation) and B1 (carbamate phosphorylation) domains of *E. coli* CPS (**A**) (PDB ID: 1jdb) and human CPS I (**B**) (PDB ID: 5dou). Both bicarbonate phosphorylation domain and carbamate phosphorylation domain in *E. coli* CPS and human CPS I have very similar folds with A1 and B1 having the traditional ATP-grasp protein fold with the *N*-terminal domain (domain A) in blue to light blue, the central domain (domain B) in green, and the *C*-terminal domain (domain C) in yellow to brown. The K-loops (residues 654–662 and 1197–1205 in human CPS I and residues 237–245 and 782–790 in *E. coli* CPS) are corresponding to the ATP binding loops in ATP-grasp proteins. In comparison to the traditional ATP-grasp proteins, both the carbonate and carbamate phosphorylation domains have an extra domain (shown in red), which hosts a T-loop (residues 777–793 and 1314–1330 in human CPS I, residue 362–378 and 895–911 in *E. coli* CPS). The bound ADP shown in stick model is located at the cleft between domains B and C, and covered by the T-loop of the extra domain.

*E. coli* CPS belongs to the CPS II family and is one of the best studied CPSs, composed of a 40-kDa glutaminase (CarA, GLN) subunit and a 120 kDa synthase (CarB, SYN) subunit [28]. The CarA subunit catalyzes the hydrolysis of glutamine to generate ammonia, which transfers to the CarB subunit. The CarB subunit consists of two homologous domains, CPS.A and CPS.B. The amino half domain catalyzes the formation of carbamate from ATP, bicarbonate, and ammonia, while the carboxyl half domain catalyzes the formation of CP from the second ATP and carbamate [29,30,31]. CPS.A and CPS.B are divided further into two subdomains, respectively, as bicarbonate phosphorylation domain (A1), integrating domain (A2) and carbamate phosphorylation domain (B1), allosteric domain (B2) [29,32]. The structure of *E. coli* CPS demonstrates that A1 and B1 have nearly identical folds while A2 and B2 have distinctly different tertiary structures [7]. The active sites are located at the A1 and B1 subdomains and the regulator-binding site is at the B2 subdomain. The structures of A1 and B1 belong to a superfamily termed “ATP-grasp” fold family that has more than 31 member proteins [5,33,34,35] (Figure 2). The ATP-grasp fold consists of two α + β domains that “grasp” ATP between them and members of the family typically have three subdomains termed A, B and C domains. All members of the ATP-grasp family catalyze an ATP-dependent ligation of a carboxyl group carbon of a substrate to an amino or imino group nitrogen of a second substrate. The substrates are quite diverse; the first substrate can take the form of a protein, a short peptide, an amino acid, or even a small chemical such as an organic or inorganic acid (carbonic, malic, succinic, and citric) whereas the second substrate can be an amino acid (Glu, Gly, Val, D-Ala, and Tyr), a thiol group (CoA), a biotinylated protein, or even a small molecule such as ammonia [34]. The catalytic mechanism of most members of the ATP-grasp family can be broken into two partial reactions, the reaction of the first substrate with ATP to form acylphosphate and the reaction of acylphosphate with the second substrate to form product. In these cases, members of the ATP-grasp family act as ligases. However, some members of the ATP grasp family do not have the second substrate and the acylphosphate is its product. In this case, these members act as kinases [35]. Remarkably, CPS combines ligase and kinase reactions into one enzyme with the A1 domain for the ligase reaction using bicarbonate as the first substrate and ammonia as the second substrate to form carbamate intermediate and the B1 domain for the kinase reaction using carbamate as the first substrate only to form the final product CP. Ammonia and carbamate are labile chemicals. Thus, a 96 Å long intermolecular channel is present in *E. coli* CPS to connect the active site of GLN domain and the active sites of the CPS.A and CPS.B domains to allow ammonia to transfer from GLN to the CPS.A domain and carbamate from the CPS.A to the CPS.B domain without equilibrating with the bulky solvent [7]. Similar intermolecular channels for ammonia and carbamate can be found in many enzymes [24,36].

Because of the essential role of CPS I in the urea cycle, human CPS I is also one of enzymes being intensely investigated [37,38,39]. Recently, the structures of human CPS I with and without the presence of NAG were determined [6,40], demonstrating that binding of NAG in the allosteric domain B2 (termed L4 in human CPS I structure) causes dramatic conformational changes of CPS I to re-shape both phosphorylation active sites in domains A1 and B1 (termed L1 and L3, respectively, in human CPS I structure) and the intramolecular tunnel for carbamate migration between them. The structures clearly show that CPS I can be in two distinctly different conformations as the inactive or active state upon the absence and presence of NAG and demonstrate the detailed molecular mechanism for NAG’s role as a switch to turn on/off the CPS I activity and eventually the ureagenesis.

The full length CPS can be composed of a single peptide chain such as CPS I and CPS III, two peptide chains such as CPS II, or even more peptide chains. In *Aquifex aeolicus*, CPS is encoded with the split genes for separate CPS.A, CPS.B, and GLN [41]. The isolated CPS.A and CPS.B subunits can catalyze both ATP-dependent partial reactions: the activation of bicarbonate to form carbamate and the phosphorylation of carbamate to form CP. Mixtures of equimolar amounts of CPS.A, CPS.B, and GLN will form a single complex with a molecular weight of 171 KDa able to catalyze the full glutamine dependent CP synthesis reaction. Even though the separated CPS.A and CPS.B are required by most CPS for the full three-step production of CP, it is not essential. Recently, a smallest CPS with a molecular weight of only 41 KDa was identified in human gut archaeon *Methanobrevibacter smithii*. The functional unit of this small CPS is a dimer corresponding to the two synthetase domains of the full-length CPS that catalyze the full three-step reaction [42]. It is now clear that the full length CPSs have arisen by gene duplication, translocation, and fusion of an ancestral ATP grasp fold kinase followed by the independent mutation of the domains for more specialized functions and the acquisition of a glutaminase able to use glutamine as a nitrogen-donating substrate [43,44,45,46]. 

### 3.2. Production of CP Using Carbamate Kinase

The formation of CP from ammonia (or glutamine), bicarbonate, and ATP in most organisms uses the above irreversible three-step reaction. However, CP can also be formed directly from carbamate and ATP in a reaction catalyzed by an entirely different enzyme, carbamate kinase (CK). This enzyme can substitute in vivo for CPS [47]. Thus, in some archaea such as *P. abyssi* and *Thermococcus kodakarensis*, in which no canonical CPS genes are identified, CK acts as the source of CP for anabolic purposes and therefore could be called CK-like CPS [9,10,11,12,48,49,50]. Since the reaction is reversible, the CP synthesis requires the presence of high concentrations of ammonia, which reacts with bicarbonate to form carbamate non-enzymatically, the substrate for CK-like CPS. Since CK-like CPS plays an anabolic role in vivo to produce CP needed for the synthesis of pyrimidine and arginine, we will term this CP-like CPS as anabolic CK, in order to distinguish it from closely related carbamate kinases (termed catabolic CK), which use CP generated from catabolic transcarbamylases to make ATP [10]. In many hyperthermophillic archaea such as *P. abyssi* and *T. kodakarensis*, the sequence of the classical CPS in the genome could not be identified, implying that this anabolic CK plays a functional role in vivo in making CP, rather than in using CP. Furthermore, kinetics evidence demonstrated that *P. furiosus* CK with both OTCase and ATCase is involved in the channeling of thermolabile CP to conform its anabolic role [51].

The high sequence identity of CKs of *P. furiosus* and *P. abyssi* (276 of the 314 residues are identical, with 31 of the 38 substitutions being conservative replacements) indicates that the *P. furiosus* protein [10] should be a good model for the structure of *P. abyssi* anabolic CK. The structure of this CK is similar to the structures of catabolic CKs (Figure 1), which uses CP to make ATP in the catabolic pathway [49,52,53,54], even though they should play different biological roles in vivo. The general fold of anabolic CK belongs to the family of amino acid kinases, whose members include *N*-acetyl-glutamate kinase [55], uridylate kinase [56], glutamate-5 kinase [57], the fosfomycin-inactivating kinase FomA [58], and aspartokinase [59]. Although the anabolic CK catalyzes the same reaction as the third step of reaction of the typical CPS (Section 3.1), neither the overall shape, the general fold, nor the disposition of the active sites has structural similarity between these two enzymes, contradicting earlier thoughts that both enzymes might have evolved from a common ancestral gene [43,60,61].

### 3.3. Production of CP Using Catabolic Transcarbamylases

CP can also be produced by a third class of entirely different enzymes, the catabolic transcarbamylases, in a fermentation process in microorganisms that includes some pathogens of medical interest such as *Mycoplasma penetrans* [53], *Giardia lamblia* [62], and *T. vaginalis* [16]. Catabolic transcarbamylase promotes phosphorolysis of ureido-containing compounds such as citrulline and carbamylputrescine to produce CP. Since this reaction is thermodynamically unfavorable, the reaction needs to couple with a downstream enzyme, carbamate kinase (catabolic CK). Gene context analysis indicates that most of the catabolic transcarbamylase genes are located in the vicinity of the carbamate kinase gene [63,64,65,66,67,68,69,70] and further confirmed the characteristic co-transcription of these two genes, which might be used to distinguish the catabolic transcarbamylase from anabolic transcarbamylase [16].

The best-known catabolic transcarbamylase is the catabolic ornithine transcarbamylase (OTCase) in the arginine deiminase pathway. The function and structures for catabolic OTCase in *Pseudomonas aeruginosa* have been studied in detail [4,13,14,71,72,73]. Even though anabolic and catabolic OTCase have high sequence similarity and catalyze the same reaction in opposite directions, in most organisms, distinct enzymes catalyze the arginine biosynthesis pathway and the catabolic arginine-deiminase pathway, respectively. Kinetically, catabolic OTCase shows highly cooperative CP binding, and AMP, CMP, and inorganic phosphate are activators of the enzyme [71]. The structures of catabolic OTCase from *P. aeruginosa* [72], *M. penetrans* [53], and *Lactobacillus hilgardii* [73] show that most catabolic OTCases have higher oligomeric structures such as a dodecamer or hexamer, in contrast to the trimeric structure of anabolic OTCase [16]. However, catabolic OTCase from *G. lamblia* is an exception since it functions as a trimer [74].

The putrescine transcarbamylase (PTCase) has also been known for many years and plays a catabolic role in the agmatine deiminase pathway using agmatine, the decarboxylated analogue of arginine, as a fermentative source of ATP [70,75,76]. Interestingly, PTCase uses a trimer as its functional molecular machinery in contrast to the catabolic OTCase, which usually assembles into a higher oligomer [16,64,77]. PTCase is unique as it uses the extra *C*-terminal helix to stabilize the trimer and prevent higher order oligomerization [64,77].

Besides the above two known catabolic transcarbamylases, CP can be generated using other catabolic transcarbamylases. It was believed that an oxamate transcarbamylase is involved in using oxalurate, a degradation product of purine, as a fermentative source to generate CP for producing ATP [78,79,80,81]. An *ygeW* gene encoded transcarbamylase in *E. coli* was proposed to catalyze this reaction [80]. However, recent studies with the recombinant protein could not confirm the oxamate transcarbamylase activity [63]. Even though the exact biological function for the *ygeW* encoded transcarbamylase still remain elusive, its biological function is most likely to be a catabolic transcarbamylase to produce CP for generating ATP. In a recent publication, a new alternative route for purine catabolism has been described, and a novel ureidoglycine transcarbamylase that uses allantoate to generate CP was identified and confirmed experimentally [68], demonstrating the need for continued investigation into the kinds of ureido-containing compounds that microorganisms can use as the energy source to make CP for ATP.

## 4. CP as Carbamyl Group Donor

CP has been known for more than 60 years to be a source of the carbamyl group that is incorporated into organic molecules in many forms of life [1,3]. The carbamyl group can be added onto either a nitrogen- or an oxygen-containing functional group, or even groups containing sulfur atoms. Very different enzymes catalyze the reactions in these carbamylation reactions. Since carbamylation of the nitrogen group is involved in several important pathways such as the arginine biosynthetic pathway, urea cycle, and pyrimidine pathway, this group of enzymes is best understood [16].

### 4.1. Amino Nitrogen as a Carbamyl Group Acceptor

**Figure 3:**
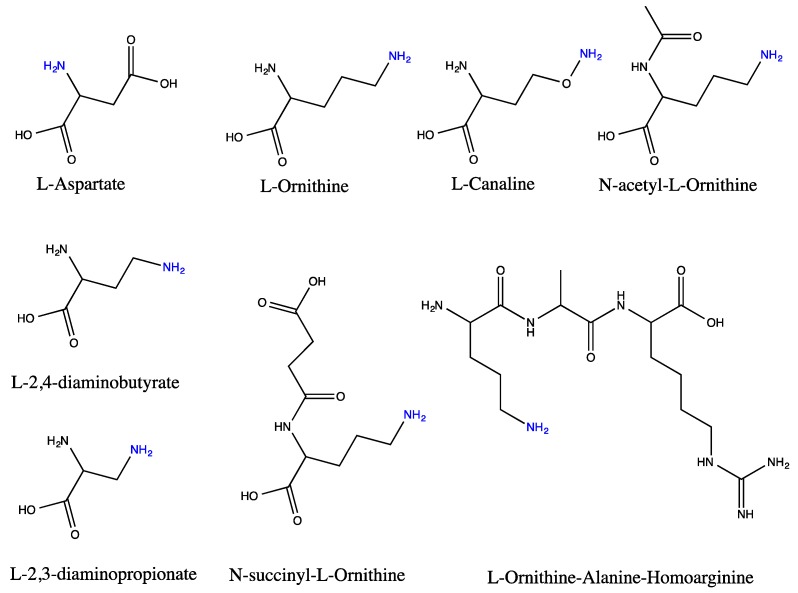
Schematic drawing of the structures of substrates of *N*-transcarbamylases. The nitrogen amino group, labeled as blue color, is the acceptor of the carbamyl group of CP during the transcarbamylation reaction catalyzed by *N*-transcarbamylases.

**Figure 4:**
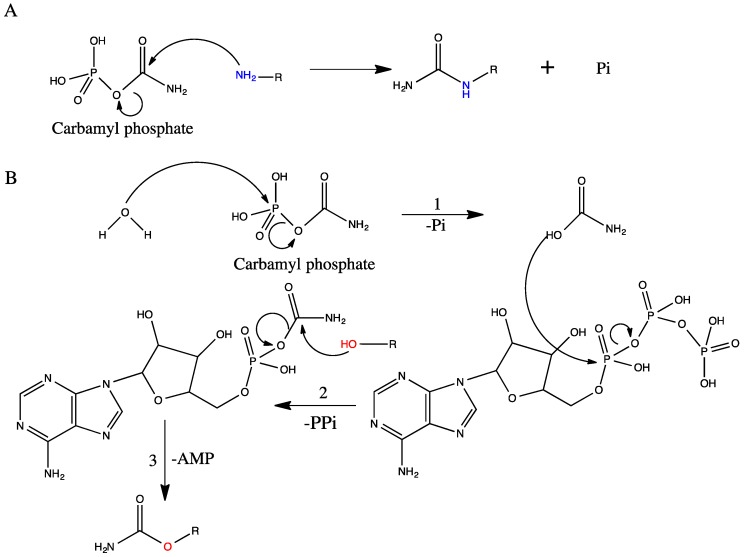
Schematic drawing of the catalytic mechanisms. (**A**) The *N-*transcarbamylases use the direct attack mechanism for the carbamyl transfer reaction from CP to the amino group; (**B**) The *O*-transcarbamylases use a three-step reaction for the carbamyl transfer from CP to the hydroxyl group via carbamyladenylate intermediate.

The amino nitrogen containing acceptor molecules include aspartate, ornithine, and various ornithine derivatives such as *N*-acetyl-, *N*-succinyl-l-ornithine or ornithine-containing peptides, and l-2,3-diamminopropionate and l-2,4-diaminobutyrate (Figure 3). The enzymes catalyzing this group of reactions are termed *N*-transcarbamylases (or transcarbamylases since they are better known than *O*-transcarbamylases, see Section 4.2). Phylogenetic analysis indicates that all members of this group of transcarbamylases can be traced back to a common ancestor gene [68,82,83]. The basic catalytic unit is a trimer with a similar protein topology to that of a subunit consisting of the *N*-terminal CP domain and the *C*-terminal acceptor-binding domain. Both domains have a αβα structure with a parallel β-sheet in the center and α helices on both sides [84]. They use a common catalytic mechanism with direct attack of the carbamyl carbon of CP by the amino nitrogen of the second substrate to form reaction product [16] (Figure 4).

#### 4.1.1. Aspartate as Acceptor

Carbamylation of the α-amino group of aspartate to form *N*-carbamyl-l-aspartate, catalyzed by ATCase, is the first reaction step in the de novo pyrimidine biosynthetic pathway [85,86]. Phylogenetic analysis classifies ATCase into two families, ATC I and ATC II [68,83]. According to the way ATCase associates with other proteins, ATCase can be divided into three classes in bacteria. ATCase in class A forms a stable dodecamer complex with the active or inactive dihydroorotase (fused or non-fused), the next enzyme in the pyrimidine biosynthetic pathway [87,88]. ATCase in class B is also a dodecamer complex but with regulatory subunits (fused or unfused) [84,89]. ATCase in Class C contains the catalytic trimer only [90]. In animals and fungi, the ATCase is fused with both CPS II and DHO (active or inactive) to form a CAD complex [44,91,92]. ATCase in plants belongs to Class C with the catalytic trimer only, but is sensitive to allosteric effectors [93,94].

Many crystal structures of ATCase with or without the associated proteins have been determined [16]. ATCase structures from *E. coli*, which represented the ATCase in class B, were studied in most detail as a model of allosteric enzymes [84,86]. The whole holoenzyme consists of two ATCase trimers at polar positions and three regulatory dimers at equatorial positions forming a dodecamer structure. Two significantly different conformations, T and R states, were identified. Either substrate or nucleotide binding alters the conformation by shifting the equilibrium between T and R states. The structure of ATCase complexed with DHO, which represents the ATCase in class A, was also resolved from *A. aeolicus* [95]. The dodecamer is arranged in such a way that two ATCase trimers are located at the two polar ends and three dihydroorotase dimers at the equator to form a hollow reactor with an internal reaction chamber about 60 Å in diameter. A similar structural arrangement to that of the core scaffold of CAD was recently proposed based on the human and fungus DHO and ATCase structural studies [96].

#### 4.1.2. Ornithine and Other Ornithine Derivatives as Acceptors

The anabolic ornithine transcarbamylase catalyzes the transfer of the carbamyl group from CP to the δ-amino group of ornithine to form citrulline in the urea cycle and arginine biosynthetic pathway. Several alternative arginine biosynthetic pathways were recently identified to demonstrate that different ornithine derivatives such as *N*-acetyl-ornithine, *N*-succinyl-ornithine, and even ornithine containing tripeptides could be the acceptor of the carbamyl group of CP [97,98,99,100]. In plants, canaline, an analogue of ornithine, can also be used as the acceptor of the carbamyl group in the canavanine biosynthetic pathway using a similar route to that for arginine synthesis [101,102].

Many crystal structures of anabolic transcarbamylases involved in the urea cycle and/or arginine biosynthetic pathway were determined [16]. Their biological functional unit is a trimer in contrast to the catabolic OTCase, whose molecular unit is usually in a higher oligomer. Interestingly, two different folding structures, one with 3_1_ trefoil knot *N*-acetylornithine and *N*-succinylornithine transcarbamylase and one without with OTCase, were identified [99,100,103].

#### 4.1.3. l-2,3-diaminopropionate and l-2,4-diaminobutyrate as Acceptor

In the biosynthesis of the antibiotics, viomycin, capreomycins, tuberactinomycines, and zwittermicin A, one reaction step involves the transfer of carbamyl group from CP to l-2,3-diaminopropionate to form β-ureidoalanine [19,20,104]. Similarly, a reaction to transfer the carbamyl group from CP to l-2,4-diaminobutyrate was found to be involved in the biosynthesis of padanamide A [17]. The enzymes that catalyze these reactions were termed l-2,3-diaminopropionate and l-2,4-diaminobutyrate transcarbamylase (DPTCase and DBTCase), respectively. Even though the structures of DPTCase and DBTCase have not been determined, bioinformatics analysis of their sequences clearly demonstrated that they closely resemble anabolic OTCase with a similar three-dimensional structural fold and a similar catalytic mechanism. Both DPTCase and DBTCase have SXRTR and HPXQ common CP binding motifs similar to other transcarbamylases. However, unlike OTCase that has conserved DXXXSMG and HCLP ornithine binding motifs, both DPTCase and DBTCase show deviation from these conserved motifs and are replaced by TRWQSMG and HDLP in DPTCase, and S/TRWQTTG and HDLP in DBTCase, respectively [16].

### 4.2. Hydroxyl Oxygen as a Carbamyl Group Acceptor

**Figure 5:**
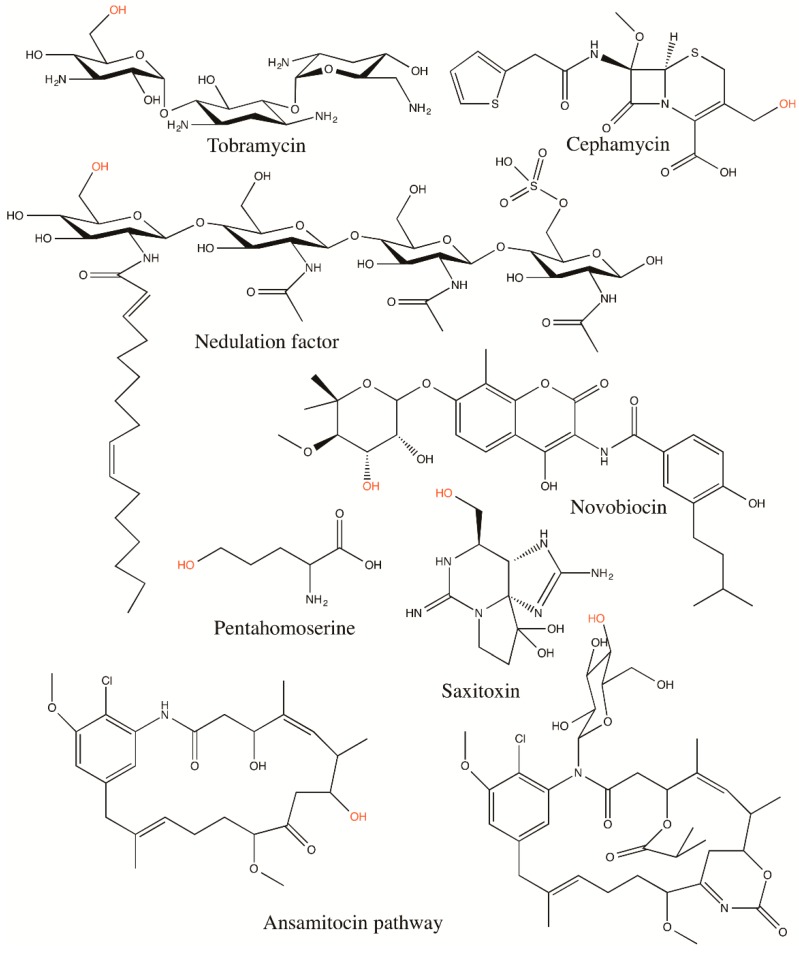
Schematic drawing of the structures of substrates of *O*-transcarbamylases. The oxygen hydroxyl group, labeled as red color, is the acceptor of the carbamyl group of CP during the transcarbamylation reaction catalyzed by *O*-transcarbamylases. In the ansamitocin biosynthetic pathway, carbamyl transfer reactions occur in two different positions, one on the backbone ring and one on the sugar group, but are catalyzed by the same *O*-transcarbamylase, Asm21.

The transfer of the carbamyl group of CP to the hydroxyl group of substrates (*O*-carbamylation) has been observed in the biosynthesis of a variety of secondary metabolites, including antibiotics such as cephalomycin [105], novobiocin [106], concanamycin A [107], ansamitocin and its derivatives [21,108], tobramycin [109], polyoxin [110], carbamyl-albicidin [23], and nebramycin [111], as well as rhizobial nodulation (Nod) factors [112] and saxitoxin [113]. In general, the substrates for *O*-transcarbamylases are much larger and more diverse than those of *N*-transcarbamylases (Figure 5). They can be divided into two types of substrates, a hydroxyl group on the sugar moiety or a non-sugar moiety.

Sequence analysis for the enzymes involved in the *O*-carbamylation indicate that they all belong to a broad class of enzymes, designated as CmcH/NodU CTases [114]. The enzymes consist of two domains: (1) the *C*-terminal YrdC-like domain which catalyzes the decomposition of CP to carbamate and phosphate, the carbamate then reacting with ATP to form the carbamyladenylate intermediate and pyrophosphate; and (2) the *N*-terminal Kae1-like domain which catalyzes the transfer of the carbamyl group from carbamyladenylate to the hydroxyl group of the substrates [22]. Interestingly, an intermolecular about 20 Å long links the carbamyladenylation to the carbamyltransfer sites to allow the relocation of the carbamyladenylate intermediate. The reaction mechanism of *O*-transcarbamylase via the carbamyladenylate intermediate (Figure 4) differs from that of *N*-transcarbamylases, which use direct transfer of the carbamyl group from CP to the substrates without involving any intermediate [16].

### 4.3. Sulfur Group as an Acceptor

**Figure 6:**
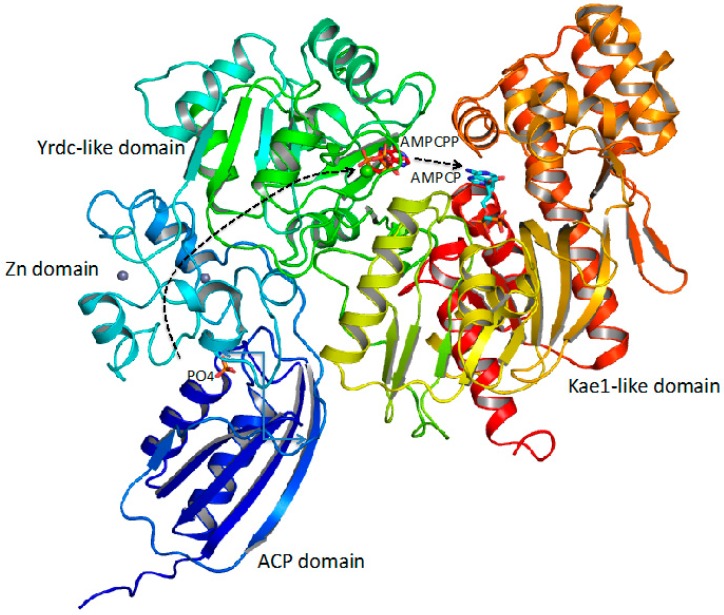
Ribbon diagram of the *S*-transcarbamylase, HypF. It catalyzes the carbamyl transfer reaction from CP to the *C*-terminal cysteine residue of HypE, another enzyme in the pathway. HypF consists of four domains: the acylphosphatase (ACP) domain, Zn finger-like domain, YrdC-like domain, and Kae1-like domain, which were colored as blue, light-blue, green and yellow to red, respectively. HypF uses the same three-step reaction mechanism for carbamyl transfer reaction as *O*-transcarbamylases*.* The bound PO_4_, AMPCPP and AMPCP, shown in stick models, mark the active sites 1, 2, and 3, respectively. The carbamate generated in the active site 1 will migrate towards the active 2 where it reacts with ATP to form carbamyladenylate via an intramolecular tunnel, then carbamyladenylate moves to the active site 3 to react with the substrate to form product.

In a key step of [NiFe]-hydrogenase complex biosynthesis, an enzyme, termed HypF, involves the catalysis of the carbamylation of the *C*-terminal cysteine residue of HypE, another enzyme in the pathway [115,116]. HypF consists of four domains: the acylphosphatase (ACP) domain, Zn finger-like domain, YrdC-like domain, and Kae1-like domain [117] (Figure 6). In comparison to the sequences of *O*-transcarbamylases, the consecutively fused YrdC-like domain and Kae1-like domain are arranged in reversed order with the YrdC-like domain in the *N*-terminal end and the Kae1-like domain in the *C*-terminal end. Furthermore, HypE employs two extra domains, ACP and Zn finger-like domains, to promote the decomposition of CP to carbamate and phosphate. Three active sites, which are involved in three reaction steps—decomposition of CP to carbamate, carbamyadenylation, and carbamyltransferation, respectively—were identified in the HypF structure with the direct distances between the first active site to the second active, and the second active site to the third active site of ~33 Å and ~15 Å, respectively [24]. Since carbamate and carbamyladenylate are highly labile in solution, similar to the mechanism used in *E. coli* CPS and human CPS I [6,7], an intramolecular channel connects these three active sites to allow carbamate to transfer from the site of CP decomposition to the carbamyladenylation site, and carbamyladenylate to the carbamyltranslation site (Figure 6).

## 5. CP as Phosphate Group Donor for ATP Production

ATP production from CP and ADP is catalyzed by catabolic CK donating the phosphate group of CP to ADP in the final step of the microbial fermentative catabolism of arginine and agmatine [52,76,118,119], and purine [68,79,81]. These catabolic pathways can be found in Bacteria [118], Archaea [120], and amitochondral Eukarya [121,122]. It is also believed that the pathways are essential for some organisms to survive such as *G. lamblia* [123]. Given the absence of fermentation pathways in higher eukaryotes including humans and the importance of energy-generation in a number of pathogenic microorganisms including *S. mutants*, *E. faecalis*, *M. penetrans*, *G. lamblia*, and *T. vaginalis*, these pathways become attractive drug development targets for some parasitic and bacterial infections.

Several catabolic CK structures have been determined including CK from *G. lamblia* [62], *M. penetrans* [53], and *E. faecalis* [54]. All these CK structures have a similar α_3_β_8_α_4_ sandwich fold with an eight-stranded β-sheet at the center and additional α helices at both sides of the central β-sheet. The CK functions as a homodimer with the central β-sheet continuing across the dimerization interface to form a 16-stranded molecular β-sheet that spans the entire molecule (Figure 1). The structures of catabolic CK are also essentially identical to that of anabolic CK [10,49], excepting some conformational differences in the active site and positional differences of the protruding subdomain [54]. It seems that whether CK plays an anabolic or catabolic role in vivo strongly depends on the presence of other enzymes and the surrounding environment, in contrast to the situation between anabolic and catabolic OTCase, in which significant differences in the oligomeric state and kinetics properties are found. CK serves an anabolic role to make CP possibly only in thermophilic archaea that live in environments able to produce carbamate non enzymatically [11].

## 6. Protection of CP

CP is a thermally unstable chemical with a half-life of ~5 min at 37 °C and physiological pH [124]. The half-life of CP at high temperatures such as 95–100 °C, the environment for thermophilic organisms to grow, is even shorter, less than 2 s. Therefore, biological systems have developed several mechanisms to protect CP from decomposition.

**Figure 7:**
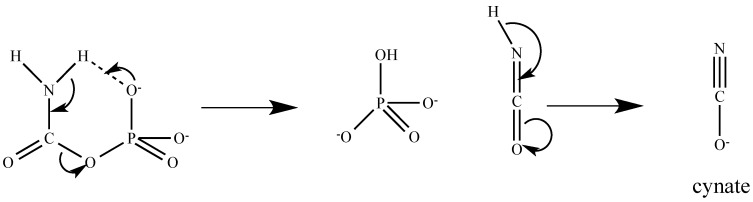
Schematic drawing for the proposed thermal decomposition of CP via an intramolecular proton transfer. In this conformation, the P-O-C-N dihedral angle is close to 0°.

The decomposition of CP has been proposed as a 2-step unimolecular elimination of cyanate via an intramolecular proton transfer to yield the phosphate anion at near neutral pH (Figure 7) [125]. The formation of a 6-membered ring structure by a hydrogen bond between the amino nitrogen and the phosphate oxygen is believed to be a critical force in driving the proton transfer. In this conformation, the P-O-C-N dihedral angle is close to 0°. It is interesting to observe that all CP bound in the enzymes have a conformation with the P-O-C-N dihedral angle close to 180° [22,99,126,127,128,129]. In this conformation, the CP is quite stable. It has been observed previously that human OTCase can protect the CP from decomposition for weeks in the active site [128]. Recent careful studies demonstrated that the binding of CP to the active sites of enzymes such as aspartate and ornithine transcarbamylases reduces the rate of thermal decomposition of CP by a factor of >5000 by restricting the CP conformation to a disfavorable geometry for decomposition [124]. In addition to the enzymatic protection of CP using the above mechanism when the second substrates are not available, most of these CP bound enzymes have high affinity for CP with a *Km* in the sub-micromole range, and a fast turnover rate for the conversion of CP to products. Furthermore, the partial channeling of CP from CPS to downstream enzymes has been suggested as part of the pyrimidine pathway in yeast [130,131], *Neurospora* [132,133], and mammals [134,135] and in the mammalian urea cycle [136,137].

Since CP is highly labile at elevated temperatures, the metabolic channeling between CPS and downstream enzymes is essential for thermophilic organisms. Kinetic experiments provide evidence for CP channeling in thermos ZO5 [138], *P. abyssi* [8], and *A. aeolicus* [139]. Co-immunoprecipitation and cross-linking experiments confirmed that the CP generating enzyme, anabolic CK, forms a functional complex with OTCase physically in *P. furiosus* for efficient CP channeling [51].

## 7. CP Accumulation and Health

**Figure 8:**
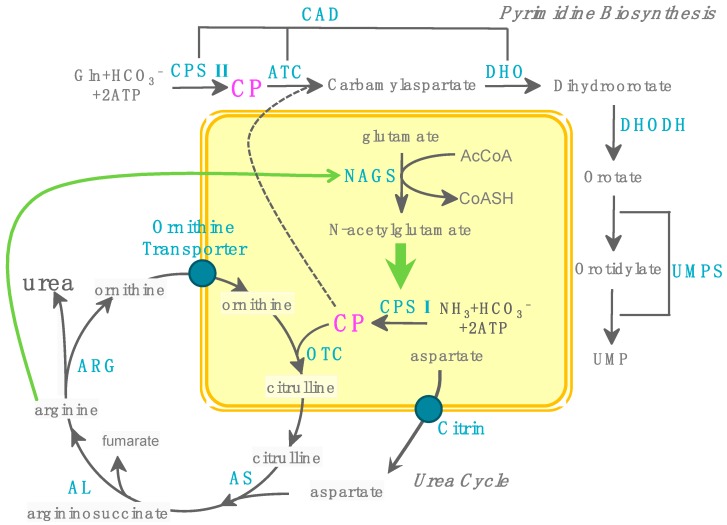
Urea cycle and pyrimidine de novo biosynthesis pathway. In mitochondria, *N*-acetyl-glutamate synthase (NAGS) generates *N*-acetylglutamate to activate CPS I to initiate the urea cycle. The CP generated by CPS I combines with ornithine to form citrulline by OTCase. Citrulline is transported out of the mitochondria to continue the last three steps by argininosuccinate synthase (AS), argininosuccinate lyase (AL), and arginase (ARG) to complete the urea cycle. Pyrimidine biosynthesis starts with the formation of CP from glutamine (Gln) by CPS II. CP reacts with aspartate to form carbamyl aspartate by ATCase (ATC), followed by cyclization by dihydroorotase (DHO) to form dihydroorotate. In humans, a single multifunctional polypeptide, CAD (CPS II, ATC and DHO) located in the cytosol, catalyzes the first three steps. Dihydroorotate dehydrogenase (DHODH) catalyzes the formation of orotate from dihydroorotate. Uridine monophosphate synthase (UMPS), a bifunctional cytosolic enzyme, completes the last two steps of pyrimidine biosynthesis to result in UMP formation. CP accumulation in the mitochondrial matrix will spill over to the cytosol to enter CAD to cause the elevation of orotate and increase pyrimidine biosynthesis. Enzymes are shown in cyan. Green arrows indicate activation of CPS1 by *N*-acetylglutamate (NAG) and NAGS by arginine. The dashed arrow indicates spillover of CP from mitochondria into cytoplasm in OTC deficiency.

In lower organisms there is generally one CPS enzyme to produce CP for both pyrimidine and arginine biosynthetic pathways, while in higher organisms there are two separate enzymes specific for each pathway. In humans, the formation of CP specific for pyrimidine synthesis occurs in the cytosol of all tissues and is catalyzed by CPS II, a part of the CAD complex, whereas CP destined for the urea cycle is formed in liver mitochondria and catalyzed by CPS I (Figure 8) [140]. The flux through the urea cycle is normally much larger than flux through the pyrimidine biosynthesis pathway so that the diversion of just a fraction of mitochondrially generated CP will substantially increase the flux through the pyrimidine biosynthesis pathway [141]. CP can accumulate within the mitochondrial matrix when the flux of CP formation by CPS I is larger than the CP consumption by OTCase. In normal conditions, CP generated from CPS I will react with ornithine to form citrulline immediately due to the much higher enzymatic activity of OTCase compared to CPS I. However, in some conditions such as OTCase deficiency or decreased supply of ornithine due to ornithine transporter (ORNT 1) deficiency, or lack of ornithine, citrulline, and arginine in the diets, CP will accumulate in the matrix. Furthermore, these conditions will also cause elevation of ammonia, which will provide more substrate to CPS I, increasing CP production for more CP accumulation.

There are two fates for accumulated CP in the matrix: decomposition or spillover into the cytoplasm, where it enters into CAD beyond the control step for pyrimidine nucleotide biosynthesis [142]. The decomposition of CP will result in the production of cyanate, a strong carbamylation agent (Figure 7). Carbamylation induced by urea-derived cyanate was one of the first post-translational modification of proteins to be described and identified in denaturation–renaturation studies of proteins with urea [143]. Carbamylation of proteins can yield both functional and structural changes in the target proteins that link to many disease conditions such as chronic kidney disease [144] and atherosclerotic vascular disease [145]. However, no disease condition has been reported related to the CP-derived cyanate production, which may be due to the lack of large amounts of cyanate accumulation in the matrix, or a protection mechanism of CP in the matrix that prevents CP decomposition to cyanate, or unrecognized disease conditions related to the CP-derived cyanate.

In comparison to the first fate of accumulated CP in the matrix, the second fate of CP is better understood. When CP enters into CAD, it will combine with aspartate to produce carbamyl aspartate by the action of ATCase, and then is further converted to orotic acid causing its elevation, eventfully resulting in nucleotide imbalance [145], a possible mechanism contributing to the cancer-promoting action of orotic acid [146]. Germline OTCase deficiency results in CP accumulation that feeds the pyrimidine pathway, causing orotic acid elevation and pyrimidine accumulation. Liver cell carcinoma has been described in an older heterozygous female OTCase deficiency patient, suggesting the possible link between CP accumulation and liver cancer [147].

Recently, it was found that CPS1 expression correlates inversely with liver kinase B1 (LKB1) activation in non-small-cell lung cancer (NSCLC) [148,149]. Surprisingly, CPS1 expression was found predominantly in the cytoplasm in these lung cancer cells, in contrast to the CPS1 only expressed inside the mitochondria in the liver and intestinal epithelial cells. It seems that cancer cells hijack CPS1 to provide an alternative pool of CP due to the increased need for pyrimidine and for maintenance of purine/pyrimidine balance. Silencing CPS I in these cancer cells will induce cell death and reduce tumor growth due to pyrimidine depletion.

## 8. Future Outlook

Since the discovery of CP in 1955, CP has been found to play important roles in many forms of life, in the synthesis of arginine and pyrimidine, removal of toxic ammonia, and production of carbamyl groups for many chemicals and phosphate groups for ATP production. The recent discovery of CP’s involvement in antibiotic production via transfer of its carbamyl group to the nitrogen amino and the oxygen hydroxyl groups of various chemicals demonstrates that the knowledge about CP is still growing [16,17,22]. CP is even found to participate in the maturation of [NiFe]-hydrogenase using CP as a source to provide the cyano ligands for the active site Fe(CN)_2_ moiety [24]. In comparison to the *N*-transcarbamylases, the structure and function of *O*-transcarbamylases are less studied, particularly how these enzymes recognize such diverse substrates. For example, the gene *Asm21* in the biosynthetic gene cluster of ansamitocin, which encodes an *O*-transcarbamylase, catalyzes not only the carbamylation of the C-7 hydroxyl group on the ansamitocin backbone with various variations, but also the carbamylation of the C-4 hydroxyl group of the *N*-β-D-glucosyl moiety of ansamitocinoside [21]. In order to understand its recognition mechanism, the elucidation of structures complexed with its various substrates is essential.

CP is produced using three very different enzymes. Among them, the ATP-grasp fold based enzyme is the most popular one for all forms of life to make CP for essential arginine/urea and pyrimidine biosynthetic pathways. The catalytic mechanisms using the three steps of partial reaction for generating CP are now well understood. The molecular mechanism for NAG activation of human CPS I has been elucidated via the structural determination of human CPS I in the absence and presence of NAG [6]. However, the allosteric molecular mechanism for UMP induced inhibition of CPS II still remains obscure because structures of *E. coli* CPS II that were determined are all in the active form similar to that of CPS I in the presence of NAG. Whether the conformation of the inactive form of CPS II exists and is similar to that of CPS I remains to be established.

Currently, the three catabolic transcarbamylases, ornithine, putrescine, and ureidoglycine transcarbamylase, which use ureido-containing compounds, citrulline, agmatine, and allantoate, as energy sources to generate CP have been firmly established. It cannot be ruled out that there are other catabolic transcarbamylases that use other different ureido-containing chemicals as substrates. The molecular identity of oxamate transcarbamylase using oxalurate as an energy source remains to be elucidated even though earlier experiments indicated that such catabolic transcarbamylase exists. Finally, the true substrate for *ygeW* encoded catabolic transcarbamylase needs to be established.

As an essential metabolite in the urea cycle, lack of or decreased CP production due to CPS I deficiency [150], lack of the activator NAG due to *N*-acetyl-glutamate synthase (NAGS) deficiency [151], or decreased supply of carbon dioxide due to carbonic anhydrase VA deficiency [152] will disrupt the urea cycle resulting in hyperammonemia. However, many disease conditions will cause the accumulation of CP. Effects of accumulation of the energy-rich and labile CP on human health is poorly understood. Furthermore, more detailed studies are certainly warranted to understand the relationship between CP and cancers as many studies demonstrate that cancer cells might hijack CPS I, probably NAGS as well, in order to meet the increased need for CP in cancer cells.
